# HEALTH RELATED SOCIAL NEEDS AND TREATMENT PREFERENCES OF OLDER ADULTS WITH SERIOUS ILLNESS

**DOI:** 10.1093/geroni/igad104.3382

**Published:** 2023-12-21

**Authors:** Amy Jackson, Joan Carpenter

**Affiliations:** University of Maryland, College Park, Maryland, United States; University of Maryland School of Nursing, Baltimore, Maryland, United States

## Abstract

The Centers for Medicare & Medicaid Services’ (CMS) health related social needs (HRSNs) include housing instability, food insecurity, transportation, utility difficulties, and interpersonal violence. HRSNs shape the experiences and outcomes of seriously ill older adults, influencing their access and ability to choose preferred treatments —from the time of diagnosis until the end of life. Little research has explored disparities in healthcare experiences related to unmet HRSNs and treatment choices, including palliative and end of life care. The purpose of this integrative review is to explore the literature regarding unmet HRSNs and provide research, practice, and policy considerations for investigators, clinicians, and payers regarding HRSNs and their impact on palliative and end of life treatment decisions. We searched PubMed, Embase, CINAHL, PsychINFO, Scopus, and Google Scholar using keywords related to HRSNs and palliative and end of life care. We identified 38 articles between 1998 and 2023 within the United States. Of the 38 articles, housing instability was the most often addressed (n=36), followed by transportation (n=1) and interpersonal violence (n=1). None of the articles explored food insecurity and utility difficulties related to serious illness and treatment preferences. Many of the articles describe the perspectives of people experiencing homelessness at the end of life (n =16). The current literature does not comprehensively address unmet HRSNs in relation to palliative and end of life care treatment choices. Future work must address HRSNs of seriously ill older adults to ensure equitable access to compassionate and person-centered palliative and end of life care.

